# ﻿Phylogeography of *Falagoniamexicana* Sharp, 1883 (Coleoptera, Staphylinidae, Aleocharinae)

**DOI:** 10.3897/zookeys.1156.84943

**Published:** 2023-03-29

**Authors:** Justo A. Reyes, Alejandro Espinosa de los Monteros, Quiyari J. Santiago-Jiménez

**Affiliations:** 1 Facultad de Biología-Xalapa, Universidad Veracruzana, Zona Universitaria, Circuito Gonzalo Aguirre Beltrán s/n, Xalapa, Veracruz, 91090, Mexico Universidad Veracruzana Xalapa Mexico; 2 Departamento de Biología Evolutiva, Laboratorio de Sistemática Filogenética, INECOL, Carretera Antigua a Coatepec No. 351, Xalapa, Veracruz, 91070, Mexico Departamento de Biología Evolutiva, Laboratorio de Sistemática Filogenética, INECOL Xalapa Mexico

**Keywords:** Cytochrome oxidase I, “false Lomechusini”, Mesoamerica, mtDNA

## Abstract

*Falagoniamexicana* is an aleocharine distributed from northern Mexico to Guatemala and El Salvador. It is associated with *Attamexicana* ants and lives within their piles of waste or external debris. The phylogeography and historical demography of 18 populations from Mexico, Guatemala, and El Salvador were studied. The data set encompasses a 472 bp fragment of the COI. Results suggest that *F.mexicana* was originated during Middle Pliocene (ca. 0.5 Mya), starting its diversification at the Upper Pleistocene and Holocene. Populations were recovered forming at least four main lineages, with a significant phylogeographic structure. Evidence of contemporary restricted gene flow was found among populations. The historical demography suggests that the geographic structure is due to recent physical barriers (e.g., Isthmus of Tehuantepec) rather than ancient geological events. Also, recent geological and volcanic events in the east of the Trans-Mexican Volcanic Belt and the Sierra Madre Oriental might be responsible for the restricted gene flow among populations. Skyline-plot analyses suggested that a demographic expansion event took place at the end of the Late Quaternary glacial-interglacial cycles.

## ﻿Introduction

*Falagoniamexicana* Sharp, 1883 is a species of Coleoptera that belongs to the subfamily Aleocharinae (Staphylinidae) (Bouchard et al. 2011). Particularly, the species is placed within Lomechusini, a tribe distinctive for having a 4–5–5 tarsal formula, metasternal process typically longer than mesosternal process, maxillar galea and lacinia moderately to considerably elongated, mesocoxae moderately to very widely separated by broad meso- and metaventral processes (Seevers 1965, 1978), among other characters. Several Lomechusini species are associated with termites and ants (Navarrete-Heredia et al. 2002), as is the case of *F.mexicana*. However, some recent studies have suggested that Lomechusini is not a monophyletic group and two clades have been proposed: the “true Lomechusini” whose species show a Holarctic distribution, and the “false Lomechusini” distributed in tropical America (Elven et al. 2010, 2012). Based on molecular data *F.mexicana* has been recovered as part of the “false Lomechusini” (unpublished data).

The genus *Falagonia* Sharp, 1883 included two species: *F.mexicana* and *F.crassiventris* Sharp, 1883. Currently, *F.crassiventris* is located in the genus *Pseudofalagonia* Santiago-Jiménez, 2010; therefore, *Falagonia* is now a monotypic genus (Santiago-Jiménez 2010; Santiago-Jiménez and Espinosa de los Monteros 2016). At a phylogenetic analysis, *Falagonia* was supported by seven autapomorphies: 1) epipharynx with a spinose process on the apico-medial margin; 2) with a pair of setae on the sensory area of ligula; 3) with simple punctures on elytral disc; 4) without tuberculate punctures on elytral disc; 5) tergite IV of male with a protuberance on each side of midline; 6) tergite VIII of male without lobes on posterior margin; and 7) tergite VIII of female without lobes on posterior margin. It is important to highlight that the specimens of *F.mexicana* are very homogeneous, except for some variation in size (4–7 mm); in color (from reddish to pale yellow in the tenerals); in the length of the α-sensilla of the epipharynx, as well as the length of the spines of the spinose process on the apico-medial margin of the epipharynx. Moreover, they present secondary sexual dimorphism. Males have a protuberance on each side of the midline of tergite IV, and a protuberance on the midline of tergite VIII.

From Mexico to El Salvador, *F.mexicana* has been recorded in a wide altitude range (from 50 to 2,000 m a.s.l.). This species has been recorded in diverse types of vegetation (Santiago-Jiménez 2010). According to Challenger and Soberón (2008) those habitats correspond to tropical evergreen forests, tropical deciduous forests, temperate coniferous and broadleaf forests, cloud forests, and scrub xerophytes. These types of vegetation in Mexico tend to present warm humid climates (Af, Am, Aw, BSh) to temperate climates (Cfb, Cwa, Cwb). Records in pine and oak forests do not exceed 2,000 m a.s.l. Thus, in general, they prefer warm humid climates with dry winter (Rzedowski 1994).

In Mexico, *F.mexicana* is found in the states of Colima, Guanajuato, Guerrero, Hidalgo, Jalisco, Michoacan, Morelos, Oaxaca, Queretaro, San Luis Potosi, Sinaloa, Sonora, Tamaulipas, and Veracruz (Santiago-Jiménez 2010). This distribution corresponds to the biogeographic provinces of the Pacific, Trans-Mexican Volcanic Belt, Sierra Madre Oriental, Sierra Madre del Sur, Soconusco, and Tierras Altas de Chiapas. These regions are associated with physiographic provinces that show heterogeneous historical processes and dates of origin (e.g., de Cserna 1989; Mastretta-Yanes et al. 2015 for summaries). For instance, the most recent formation corresponds to the Trans-Mexican Volcanic Belt from the Miocene to the present day, with some stratovolcanoes forming in during the Pleistocene (Ferrari et al. 2012).

*Falagoniamexicana* lives in the debris produced by *Attamexicana* (Smith, 1858) ants. The reddish color *F.mexicana* allows it to blend in with the debris. Apparently, despite having developed wings, this species does not fly. Flight intercept traps have been placed near the debris and we have not been able to catch them. Instead, by placing pit-fall traps in the vicinity of the detritus we have obtained some specimens. The nests of *A.mexicana* with its associated debris seems to be islands in the geographic distribution of *F.mexicana*, due to their presumed inability to fly and limited long-distance movement. Therefore, geographical barriers have a great effect in reducing gene flow between its populations, as well as restrictions on its geographical distribution. Physical barriers can lead to genetic isolation when they remain for an extended period of time, which is essential for lineage divergence (Saunders et al. 1991; Avise 2000).

Apparently, both *A.mexicana* and *F.mexicana* are incapable of surviving in areas above 2,300 m a.s.l. In the case of *F.mexicana*, its septentrional distribution is limited by the Tropic of Cancer, as is the case with other insect groups (e.g., Passalidae, Reyes-Castillo 2000). However, *A.mexicana* does reach the southern United States. It has been seen in the field that *F.mexicana* prefers debris that is under vegetal cover, with more shade and humidity. Usually, *F.mexicana* is found in *A.mexicana*’s detritus, but that does not mean that every *A.mexicana* nest has *F.mexicana* associated with it, especially when the detritus is exposed to desiccation.

Recently, some studies have been carried out to understand the biogeographical patterns of the biota in Middle America (e.g., Marshall and Liebherr 2000; Daza et al. 2010; Ornelas et al. 2013). With an average altitude of 200 m a.s.l. the Isthmus of Tehuantepec has been identified as an effective barrier for gene flow (Barrier et al. 1998). Likewise, the Trans-Mexican Volcanic Belt represents a biogeographical barrier (Marshall and Liebherr 2000) and a center for lineage diversification (Bryson et al. 2018). The use of coalescent and geographic structure models based on molecular data can be useful to test historical demographic scenarios where hypothesis of vicariance and dispersion can be contrasted (Knowles and Carstens 2007; Daza et al. 2010). Therefore, *F.mexicana* is an interesting model to study the relationship between genealogy and geography. The objectives of the present study are to estimate the date of the appearance of *Falagoniamexicana*, to evaluate the phylogenetic structure of *F.mexicana*, and to correlate the historic events that explain the divergence processes within the lineage.

## ﻿Materials and methods

**Figure 1.**
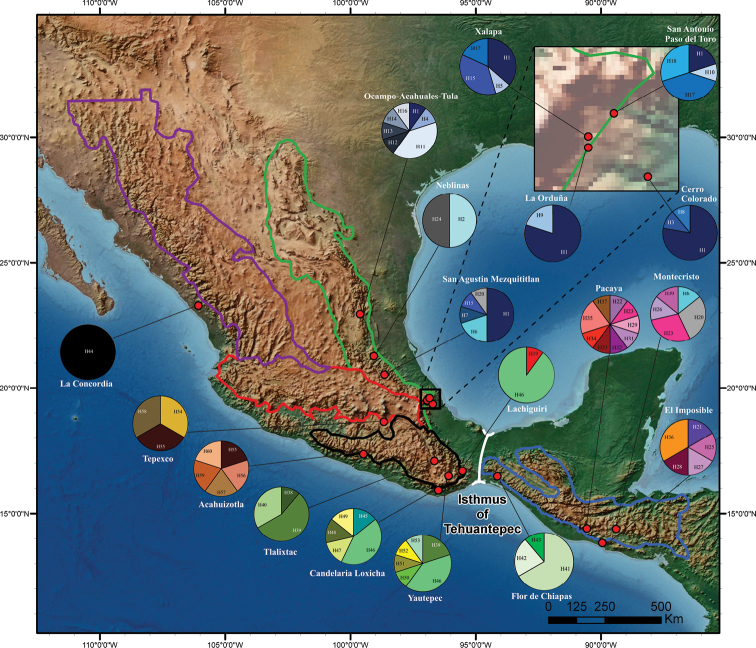
Distribution map showing the localities where individuals of *Falagoniamexicana* were sampled. Pie charts represent haplotypes sampled in each locality. Color on map indicates altitudinal changes every 500 m a.s.l. The polygons represent the main biogeographical regions: Sierra Madre del Sur (black), Sierra Madre Occidental (purple), Sierra Madre Oriental (green), Tierras Altas de Chiapas (blue), and Trans-Mexican Volcanic Belt (red). The white line represents the Isthmus of Tehuantepec.

The sampling was carried out in 18 localities from El Salvador, Guatemala, and Mexico (Fig. 1, Table 1). The collection sites were selected based on previous records and potential suitable sites. Potential localities were identified based on their environmental and geographic conditions. This sampling covers most of the species’ distribution. Field collections were carried out by direct collection on debris of *A.mexicana* using an aspirator. After its collection, the specimens were placed in 96% ethanol and stored at -20 °C. The material was deposited at the Phylogenetic Systematics Laboratory (INECOL, Xalapa).

**Table 1.** Collection sites for samples of *Falagoniamexicana* examined in the present study (*n* = 139).

Country	Locality	Region	*n*	Altitude (m a.s.l.)	Coordinates	Haplotypes
El Salvador	SAN: Montecristo	TAC	7	1340	14°23.18'N, 89°24.01'W	H6(1), H20(2), H23(2), H26(1), H30(1)
El Salvador	AHU: El Imposible	TAC	6	810	13°49.65'N, 89°56.84'W	H21(1), H25(1), H27(1), H28(1), H36(2)
Guatemala	GUA: Pacaya	TAC	10	1350	14°24.22'N, 90°33.61'W	H22(1), H23(1), H29(1), H31(1), H32(1), H33(1), H34(1), H35(2), H37(1)
Mexico	GUE: Acahuizotla	SMS	5	1100	17°21.32'N, 99°28.68'W	H55(1), H56(1), H57(1), H59(1), H60(1)
Mexico	HGO: San Agustin Mezquititlan	SMO	10	1350	20°31.90'N, 98°38.40'W	H1(5), H6(2), H7(1), H15(1), H20(1)
Mexico	OAX: Candelaria Loxicha	SMS	7	500	15°55.42'N, 96°29.33'W	H45(1), H46(3), H47(1), H48(1), H49(1)
Mexico	OAX: Flor de Chiapas	SOC	9	1130	16°28.48'N, 94°08.13'W	H41(6), H42(2), H43(1)
Mexico	OAX: Lachiguiri	SMS	10	810	16°41.39'N, 95°31.72'W	H19(1), H46(9)
Mexico	OAX: Tlalixtac	SMS: VCO	9	1590	17°04.86'N, 96°39.03'W	H38(1), H39(5), H40(3)
Mexico	OAX: Yautepec	SMS: VCO	10	760	16°29.84'N, 96°05.56'W	H39(2), H46(4), H50(1), H51(1), H52(1), H53(1)
Mexico	PUE: Tepexco	TMV	3	1290	18°39.03'N, 98°39.88'W	H54(1), H55(1), H58(1)
Mexico	QUE: Neblinas	SMO	2	650	21°16.08'N, 99°03.98'W	H2(1), H24(1)
Mexico	SIN: La Concordia	PAC	1	130	23°17.27'N, 106°03.98'W	H44(1)
Mexico	TAM: Ocampo-Acahuales-Tula	SMO	10	1030	22°56.17'N, 99°31.10'W	H1(1), H4(1), H11(4), H12(1), H13(1), H14(1), H16(1)
Mexico	VER: Cerro Colorado	SMO	9	520	19°21.15'N, 96°42.69'W	H1(7), H3(1), H8(1)
Mexico	VER: La Orduña	SMO	10	1210	19°27.64'N, 96°56.08'W	H1(8), H9(2)
Mexico	VER: San Antonio Paso del Toro	SMO	10	1010	19°35.28'N, 96°50.27'W	H1(2), H10(1), H17(4), H18(3)
Mexico	VER: Xalapa	SMO	11	1360	19°30.17'N, 96°56.05'W	H1(4), H5(1), H15(4), H17(2)

AHU Ahuachapan, SAN Santa Ana, GUA Guatemala, GUE Guerrero, HGO Hidalgo, OAX Oaxaca, PUE Puebla, QUE Queretaro, SIN Sinaloa, TAM Tamaulipas, VER Veracruz, TAC Tierras Altas de Chiapas, SMS Sierra Madre del Sur, SMO Sierra Madre Oriental, SOC Soconusco, TMV Trans-Mexican Volcanic Belt, PAC Pacific, VCO Valles Centrales de Oaxaca.

From total genomic DNA, we obtained the sequence for a fragment of 472 base pairs (bp) of the Cytochrome Oxidase subunit I (COI) mitochondrial gene. This fragment is located between positions 250 and 720 of the COI gene. This gene was selected since it presents an adequate mutation rate for this kind of studies (Avise 2000). Sequences present extensive intraspecific polymorphisms, providing information between individuals. In addition, this molecular marker has been a useful tool for species’ identification (Avise 2000). DNA extraction was performed using the kit Tissue & Insect DNA MicroPrep (Zymo Research Corp., Irvine, CA), following the protocol suggested by the manufacturer. DNA was extracted for amplification from 139 specimens. Amplification was performed using the S1718 (Normark 1996) and NANCY (Simon et al. 1994) primers designed for Coleoptera. PCR mix consisted of 5 µL of DNA, 3 µL of 5X Buffer, 3 µL of MgCl2 (25mM), 3 µL of dNTP’s (8mM), 3 µL of each primer (10mM), 0.2 µL of Taq polymerase (5 U / µL) and 9.8 µL of ddH2O adding to a final volume of 30 µL. The PCR protocol consisted of an initial denaturation for 180 s at 94 °C; 35 cycles of 30 s at 94 °C as denaturing temperature, 30 s at 45 °C for primer annealing and 90 s at 74 °C for extension; followed by one final extension cycle of 300 s at 72 °C. PCR products were visualized in a 1% agarose gel stained with ethidium bromide and purified using the GeneJET Genomic DNA Purification Kit (Thermo Fisher Scientific Inc., Waltham, MA). The PCR samples were sent to the Macrogen company (South Korea) for sequencing. Sequences were assembled with the software SEQUENCHER v. 5.2.4 (Gene Codes Corporation). The alignment of the sequences was trivial; therefore, it was done manually using the software MEGA v. 7 (Kumar et al. 2016). All the sequences acquired for the first time were deposited in GenBank (OQ608846–OQ608984).

The identity of the sequences was corroborated by translating them into amino acids, verifying the absence of nonsense codons or intermediate stops, and by a BLAST search. Using the software PAUP v. 4.0 (Swofford 2002), the nucleotide proportion of each sequence was estimated, as well as their standard deviation and nucleotide bias. In turn, the first, second, and third positions were identified, and the same descriptors were calculated. The proportion of transitions and transversions were also calculated.

To identify genetically congruent geographic regions, a spatial analysis of molecular variance (SAMOVA) was conducted. This was performed with the aid of the software SAMOVA v. 1 (Dupanloup et al. 2002), using 100 banding steps to obtain the fixation index *F*_CT_ (Excoffier et al. 1992). Likewise, a non-hierarchical analysis of molecular variance (AMOVA) was carried out including every population, followed by an analysis between the statistically significant regions identified by the SAMOVA. This was conducted with 1000 permutations using the software ARLEQUIN v. 3.5 (Excoffier and Lischer 2010). A Mantel test was performed, based on the combined data set, to detect possible isolation by distance. Genetic variation within and between populations was evaluated using descriptive empirical values, such as number of haplotypes (*H*), haplotype diversity (*Hd*), nucleotide diversity (π) (Nei 1987), and segregated sites (*S*) (Nei and Kumar 2000). These values were estimated using the software DNASP v. 5.10 (Librado and Rozas 2009). This analysis was done for each population, as well as for each region inferred by the SAMOVA. The haplotype network was made with the software NETWORK v. 4.5 (Bandelt et al. 1999) using the Median Joining algorithm with a weight of 10 for all characters and an epsilon of 10 as suggested by the authors. Similarly, the Maximum Parsimony post-processing tool was used to eliminate all the superfluous intermediate haplotypes and the links that were not in the shortest network.

The genealogy of the sequences was estimated with a Bayesian inference analysis, using the software MRBAYES v. 3.2.3 (Ronquist et al. 2012). The best-fit nucleotide substitution model was selected using the Akaike information criterion through the software JMODELTEST v. 2.02 (Posada 2008). A homologous sequence of *Pseudofalagonia* sp. (acc. num. OL764948.1), a species closely related to *F.mexicana*, was used as outgroup. The Markov Monte Carlo Chains (MCMC) were run for 10 million generations sampling trees and parameters every 1000 generations. The consensus tree was obtained with their respective posterior probabilities after discarding the initial 25% of the stored trees (Ronquist et al. 2012). Likewise, an inference based on the Maximum Likelihood (ML) optimality criterion was carried out using the software RAXML v. 8 (Stamatakis 2014). The robustness of the nodes recovered in the ML tree was evaluated with 500 bootstrap replications.

To date and infer possible isolation events in *F.mexicana* populations, we used the software BEAST v. 1.10.4 (Drummond et al. 2012). To optimize tree search, the topology inferred from the Maximum Likelihood analysis was used as the tree prior. The ML tree was used instead of the Bayesian one because BEAST only accepts fully resolved trees as priors. To estimate the divergence times, we used a relaxed uncorrelated clock model.

A nucleotide substitution rate of 6.311 (10^-2^ subs/site/My/lineage) for the COI was used to date the genealogy of *Falagonia*. This rate between taxa is based on the substitution rate estimated by Pons et al. (2010) for the COI gene of the coleopteran mitochondrial genome. Alternatively, a more conserve substitution rate of 2.0 (10^-2^ subs/site/My/lineage) was used (chronogram available as Suppl. material 1). This rate closely matches the standard mitochondrial arthropod clock reported by Brower (1994), and the rates calculated in studies that compared closely related species of Coleoptera (e.g., Leys et al. 2003; Balke et al. 2009; Ribera et al. 2010). The BEAST MCMC ran for 10 million generations, sampling trees and parameters every 1000 generations. The software TRACER v. 1.6 (Rambaut et al. 2014) was used to evaluate stationarity of the MCMC parameters, sample sizes (ESSs > 200) and posterior intervals spanning the 95% of the highest posterior density. This analysis was repeated four times and the trees resulting from each run were combined using the software LOGCOMBINER v. 1.10.4 (Drummond et al. 2012), after applying an initial burn-in of 25%. The evaluated nodes were those that scored a posterior probability value higher than 0.9. The inferred chronogram was viewed and edited with the software FIGTREE v. 1.4.2 (Rambaut 2008). To determine the historical demography of the populations, we conducted a neutrality test with Fu’s *F* statistics (Fu 1997). This was compared with the generalized skyline-plot analysis inferred with BEAST. The input parameters, as well as the molecular clock model, were identical to the ones used for the coalescence analysis for node dating.

## ﻿Results

The alignment of the 139 COI sequences showed 417 constant positions (88%). The 55 remaining characters correspond to variable sites (See Suppl. material 2), of which 39 of them are potentially phylogenetically informative, while the other 16 are singletons. Sequences present a 0.27 nucleotide bias and the following nucleotide percentages: A 29.8%, C 17.2%, G 14.9%, and T 38.1%. Regarding codon positions, first positions show less nucleotide bias (0.11) and the following nucleotide proportion: A 25%, C 17.6%, G 25.0%, and T 31.2%. Second positions present an intermediate bias (0.28) and the following nucleotide percentages: A 13.3%, C 28.8%, G 17.2%, and T 40.7%. Lastly, third positions show the highest nucleotide bias (0.64) and this nucleotide composition: A 50.4%, C 17.2%, G 14.9%, and T 38.1%. Regarding base substitution mutations we found a total of 48 transitions and 30 transversions, resulting in a Ts:Tv = 1.6:1 ratio. Mutation transitions were as follows: 27 A-G and 21 C-T; while transversions: 10 A-T, 10 T-G, 7 A-C and 3 C-G.

**Figure 2.**
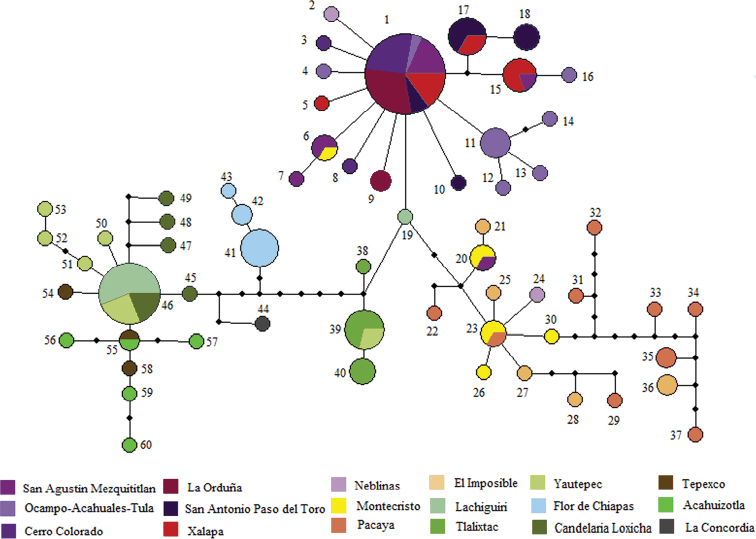
Haplotype network of *Falagoniamexicana*. The network was based on statistical parsimony for the 60 haplotypes retrieved from the COI sequences. Numbers indicate haplotype identity. Colors on network correspond to each locality as shown in the legend.

The haplotype network (Fig. 2) contains 60 haplotypes distributed among the 18 localities. Most of the localities exhibited more than one haplotype. Two main haplotypes (i.e., H1, H46) connected by eleven mutations were recovered. Most of the Sierra Madre Oriental populations had haplotype H1 individuals; while haplotype H46 is shared by individuals from the Sierra Madre del Sur, and one population from the Valles Centrales de Oaxaca. Haplotypes H6 and H20 are shared between the Sierra Madre Oriental and Tierras Altas de Chiapas regions. Haplotypes H2 and H24 collected in Neblinas (Queretaro state) were recovered in the network as the most closely related to the haplotypes from Sierra Madre Oriental and Tierras Altas de Chiapas, respectively. Haplotype H19 from Lachiguiri (Oaxaca state) is an intermediate haplotype among those of Sierra Madre Oriental, Tierras Altas de Chiapas, and Sierra Madre del Sur in Valles Centrales de Oaxaca. Individuals from Tlalixtac and Yautepec (Valles Centrales de Oaxaca) shared Haplotype H39. Haplotypes H41, H42, and H43 are endemic to Flor de Chiapas. Haplotypes H38 and H40 are endemic to Tlalixtac. Haplotypes H45, H47, H48 and H49 are endemic to Candelaria Loxicha (Oaxaca state), and haplotypes H50 to H53 to Yautepec. Haplotypes H54 to H60 were found in individuals from the Sierra Madre del Sur (Acahuizotla, Guerrero state) and the Trans-Mexican Volcanic Belt (Tepexco, Puebla state) regions. Global and local haplotype frequencies can be consulted in Suppl. material 3.

Genetic variation descriptors (Table 2) show that within populations the number of segregated sites range from 1 to 16. The highest nucleotide variation value was found in the population of Neblinas (π = 0.0149, *S* = 7). This locality had nearly 30 times the nucleotide variation found in Flor de Chiapas (π = 0.0005, *S* = 1). The lowest value for haplotype diversity was at Lachiguiri (*Hd* = 0.2), while the highest value (i.e., 1) was at Acahuizotla (Guerrero), Neblinas (Queretaro) and Tepexco (Puebla). The global Fu’s *F* value (i.e., -7.935; *p* < 0.001) is consistent with a significant demographic expansion history. However, none of the individual populations showed statistically significant values for this descriptor.

**Table 2.** Indices of genetic diversity from the sequences of the 472 bp fragment of COI from *F.mexicana*.

Population	*n*	*S*	π	S.D. π	*h*	*Hd*	S.D. *Hd*	Fu’s *F*	*p*
Flor de Chiapas	9	1	0.0005	0.0004	2	0.222	0.166	-0.263	0.342
La Orduña	10	1	0.0008	0.0003	2	0.356	0.159	0.417	0.400
Cerro Colorado	9	2	0.0011	0.0005	3	0.417	0.191	-1.081	0.196
San Antonio Paso del Toro	10	2	0.0015	0.0005	3	0.511	0.164	-0.272	0.282
Xalapa	11	3	0.0029	0.0004	4	0.764	0.083	-0.290	0.251
Tepexco	3	4	0.0059	0.0022	3	1.000	0.272	-0.341	0.416
Tlalixtac	9	4	0.0025	0.0011	3	0.639	0.126	0.645	0.329
Candelaria Loxicha	7	5	0.0041	0.0012	4	0.714	0.181	-0.324	0.274
Neblinas	2	7	0.0149	0.0075	2	1.000	0.500	1.946	0.875
Acahuizotla	5	7	0.0069	0.0016	5	1.000	0.126	-2.238	0.096
El Imposible	6	7	0.0079	0.0015	4	0.867	0.129	0.426	0.352
Ocampo-Acahuales-Tula	10	7	0.0048	0.0010	7	0.867	0.107	-3.310	0.029
San Agustin Mezquititlan	10	8	0.0044	0.0018	5	0.756	0.130	-0.760	0.201
Montecristo	7	9	0.0078	0.0024	4	0.810	0.130	0.812	0.327
Pacaya	10	11	0.0079	0.0017	7	0.867	0.107	-1.744	0.109
Lachiguiri	10	12	0.0053	0.0041	2	0.200	0.154	4.582	0.069
Yautepec	10	16	0.0127	0.0039	6	0.844	0.103	0.778	0.272
La Concordia	1	0	0.0000	–	1	0	–	–	–
Xalapa, Orduña, and San Antonio Paso del Toro	31	4	0.0020	0.0003	5	0.652	0.063	-0.977	0.159
Acahuizotla and Tepexco	8	10	0.0064	0.0015	6	0.929	0.084	-1.785	0.113
Global	139	35	0.0161	0.0007	34	0.862	0.022	-7.935	< 0.001

*n*: samples number; *S*: number of segregating sites; π: nucleotide diversity; S.D.: standard deviation; *h*: haplotype numbers; *Hd*: haplotype diversity; *p*: probability value.

The SAMOVA (*k* change from 2 to 15) showed a gradual increase in the *F*_CT_ values (Table 3). The highest *F*_CT_ was reached with 15 groups, where the genetic variability among groups reached approximately 80%. This result suggests that practically every locality is an isolated group. The only two groups recovered were: Xalapa-La Orduña-San Antonio Paso del Toro (central Veracruz state) and Acahuizotla-Tepexco. The Mantel test revealed a significant positive correlation (r = 0.3756, *p* < 0.0001), which suggests isolation by distance. The AMOVA showed also significant genetic structure (*F*_ST_ = 0.77, *P* < 0.0001). Approximately 77% of molecular variance occurs among populations, while the remaining 23% is explained by the genetic differences within populations (Table 4). Population pairwise *F*_ST_ values revealed significant differentiation between most populations (Table 5). Gene flow estimations among populations were extremely varied, fluctuating from *M* = 0.000 (i.e., between Flor de Chiapas-San Antonio Paso del Toro) to a continuous exchange of individuals between Xalapa-La Orduña, and Xalapa-Cerro Colorado.

**Table 3.** Fixation index (*F*_CT_) for population groups of *F.mexicana* recovered by the SAMOVA.

Population groups	*k*	*F*_CT_	*p*
(Xal, Ord, CCo, Mez, OAT, Neb, Imp, Mon, Pac, SAPT, Tla) (Aca, CaL, Tep, Lac, FDC, Yau, Con)	2	0.613	< 0.00001
(Xal, Ord, Cco, Mez, OAT, Neb, SAPT y Tla) (Aca, CaL, Tep, Lac, FDC, Con) (Imp, Mon, Pac)	3	0.634	< 0.00001
(Xal, Ord, Cco, Mez, OAT, Neb, SAPT, Tla) (Imp) (Aca, CaL, Tep, Lac, FDC, Yau, Con) (Mon, PAC)	4	0.620	< 0.00001
(Xal, Ord, Cco, Mez, OAT, Neb, SAPT, Tla) (Imp, Mon) (Pac) (FDC) (Aca, CaL, Tep, Lac, Yau, Con)	5	0.680	< 0.00001
(Xal, Ord, Cco, Mez, OAT, Neb, SAPT) (Tla) (Imp, Mon, Pac) (Aca, Tep, Con) (CaL, Lac, Yau) (FDC)	6	0.760	< 0.00001
(Xal, Cco, Mez, OAT, Neb, SAPT) (Ord) (Tla) (Imp, Mon, Pac) (Aca, Tep, Con) (CaL, Lac, Yau) (FDC)	7	0.741	< 0.00001
(Xal, Ord, Cco, Mez, OAT, Neb, SAPT) (Tla) (Imp, Mon) (Pac) (Aca, Tep) (CaL, Lac, Yau) (Con) (FDC)	8	0.771	< 0.00001
(Xal, Ord, Cco, Mez, OAT, Neb, SAPT) (Tla) (Imp, Mon) (Pac) (Aca, Tep) (CaL) (Lac, Yau) (FDC) (Con)	9	0.767	< 0.00001
(Xal, Ord, Cco, Mez, SAPT) (OAT, Neb) (Tla) (Imp, Mon) (Pac) (Aca, Tep) (CaL) (Lac, Yau) (Con) (FDC)	10	0.774	< 0.00001
(Xal, Ord, Cco, Mez, SAPT) (Neb) (OAT) (Tla) (Imp, Mon) (Pac) (Aca, Tep) (CaL) (Lac, Yau) (Con) (FDC)	11	0.783	< 0.00001
(Xal, Ord, Cco, Mez, SAPT) (OAT) (Neb) (Tla) (Imp, Mon) (Pac) (Aca) (Tep) (CaL) (Lac, Yau) (Con) (FDC)	12	0.781	< 0.00001
(Xal, Ord, Cco, SAPT) (Mez) (OAT) (Neb) (Tla) (Imp) (Mon) (Pac) (Aca) (Tep) (CaL, Lac, Yau) (Con) (FDC)	13	0.780	< 0.00001
(Xal, Ord, Cco, Mez, SAPT) (OAT) (Neb) (Tla) (Imp) (Mon) (Pac) (Aca) (Tep) (CaL) (Lac) (Yau) (Con) (FDC)	14	0.792	< 0.00001
(Xal, Ord, SAPT) (Cco) (Mez) (OAT) (Neb) (Tla) (Imp) (Mon) (Pac) (Aca, Tep) (CaL) (Lac) (Yau) (Con) (FDC)	15	0.794	< 0.00001

*k*: number of groups; *F*_CT_: genetic diversity between groups; *p*: p-value. Xal: Xalapa, Ord: La Orduña, CCo: Cerro Colorado, Mez: San Agustin Mezquititlan, OAT: Ocampo-Acahuales-Tula, Neb: Neblinas, Imp: El Imposible, Mon: Montecristo, Pac: Pacaya, SAPT: San Antonio Paso del Toro, Tla: Tlalixtac, Aca: Acahuizotla, CaL: Candelaria Loxicha, Tep: Tepexco, Lac: Lachiguiri, FDC: Flor de Chiapas, Yau: Yautepec, Con: La Concordia.

**Table 4.** Analysis of molecular variance (AMOVA) among the 18 populations of *F.mexicana*.

Variation Source	d. f.	Sum of squares	Variance components	Variation (%)	*F*_ST_
Among populations	17	507.30	3.75	77.03	0.77*
Within populations	121	135.39	1.12	22.97	
Total	138	642.69	4.87		

*= Significance < 0.000001.

**Table 5.** Paired comparisons of *F.mexicana* populations. Above the diagonal: Number of migrants per generation (*M*). Below the diagonal: Fixation Index (*F*_ST_).

	Xal	Ord	CCo	Mez	OAT	Neb	Aca	CaL	Imp	Mon	Pac	SAPT	Tep	Lach	Tlal	FDC	Yau	Con
**Xalapa**		inf	inf	6.100	0.713	0.252	0.028	0.026	0.139	0.214	0.214	inf	0.035	0.061	0.048	0.011	0.167	0.023
**La Orduña**	-0.01		4.578	3.102	0.523	0.152	0.014	0.015	0.113	0.178	0.193	inf	0.018	0.051	0.024	0.000	0.158	0.000
**Cerro Colorado**	-0.02	0.098		inf	1.168	0.504	0.048	0.041	0.198	0.308	0.274	4.578	0.060	0.080	0.082	0.023	0.209	0.057
**San Agustin Mezquititlan**	0.076	0.139	0.00		2.326	2.088	0.109	0.086	0.411	0.643	0.454	3.102	0.135	0.123	0.204	0.068	0.264	0.182
**Ocampo-Acahuales-Tula**	0.412*	0.489*	0.300*	0.177*		1.102	0.083	0.065	0.296	0.413	0.342	0.523	0.103	0.097	0.145	0.050	0.221	0.127
**Neblinas**	0.665	0.766	0.498	0.193	0.312		0.140	0.089	3.066	inf	814.4	0.152	0.274	0.153	0.237	0.047	0.520	1.250
**Acahuizotla**	0.947*	0.973*	0.913*	0.821*	0.858	0.781		0.192	0.106	0.133	0.145	0.014	inf	0.377	0.037	0.030	1.373	0.099
**Candelaria Loxicha**	0.951*	0.971*	0.924*	0.854*	0.885*	0.848	0.723*		0.077	0.098	0.108	0.015	0.337	inf	0.033	0.029	4.371	0.097
**El Imposible**	0.782*	0.816*	0.716*	0.549*	0.628*	0.140	0.825*	0.866*		17.01	3.169	0.113	0.144	0.112	0.150	0.056	0.266	0.197
**Montecristo**	0.700*	0.738*	0.619*	0.438*	0.548*	-0.05	0.790*	0.836*	0.029		3.625	0.178	0.179	0.137	0.246	0.081	0.319	0.277
**Pacaya**	0.701*	0.722*	0.646*	0.524*	0.594*	0.001	0.776*	0.822*	0.136	0.121		0.193	0.180	0.140	0.223	0.093	0.283	0.235
**San Antonio Paso del Toro**	-0.01	0.000	0.098	0.139	0.489*	0.766	0.973*	0.971*	0.816*	0.738*	0.722*		0.018	0.051	0.024	0.000	0.158	0.000
**Tepexco**	0.935*	0.965*	0.892*	0.787*	0.830*	0.646	-0.01	0.597	0.777	0.737*	0.736*	0.965*		0.633	0.048	0.040	4.059	0.304
**Lachiguiri**	0.892*	0.907*	0.862*	0.803*	0.837*	0.766	0.570*	-0.05	0.816*	0.785*	0.782*	0.907*	0.441		0.075	0.089	9.323	0.294
**Tlalixtac**	0.913	0.955*	0.860*	0.710*	0.775*	0.678	0.931*	0.938*	0.770*	0.670*	0.692*	0.955*	0.912*	0.869*		0.016	0.264	0.032
**Flor de Chiapas**	0.979*	1.000*	0.956*	0.880*	0.909*	0.913	0.944*	0.946*	0.900*	0.861*	0.844*	1.000*	0.927*	0.849*	0.970*		0.287	0.000
**Yautepec**	0.750*	0.760*	0.705*	0.655*	0.694*	0.490	0.267	0.103	0.653*	0.610*	0.638*	0.760*	0.110	0.051	0.654*	0.635*		3.368
**La Concordia**	0.955	1.000	0.897	0.733	0.798	0.286	0.835	0.838	0.717	0.644	0.680	1.000	0.622	0.629	0.940	1.000	0.129	

Bold values showed a significance value of *p* < 0.01. Abbreviations of the localities correspond to those used in Table 3.

**Figure 3.**
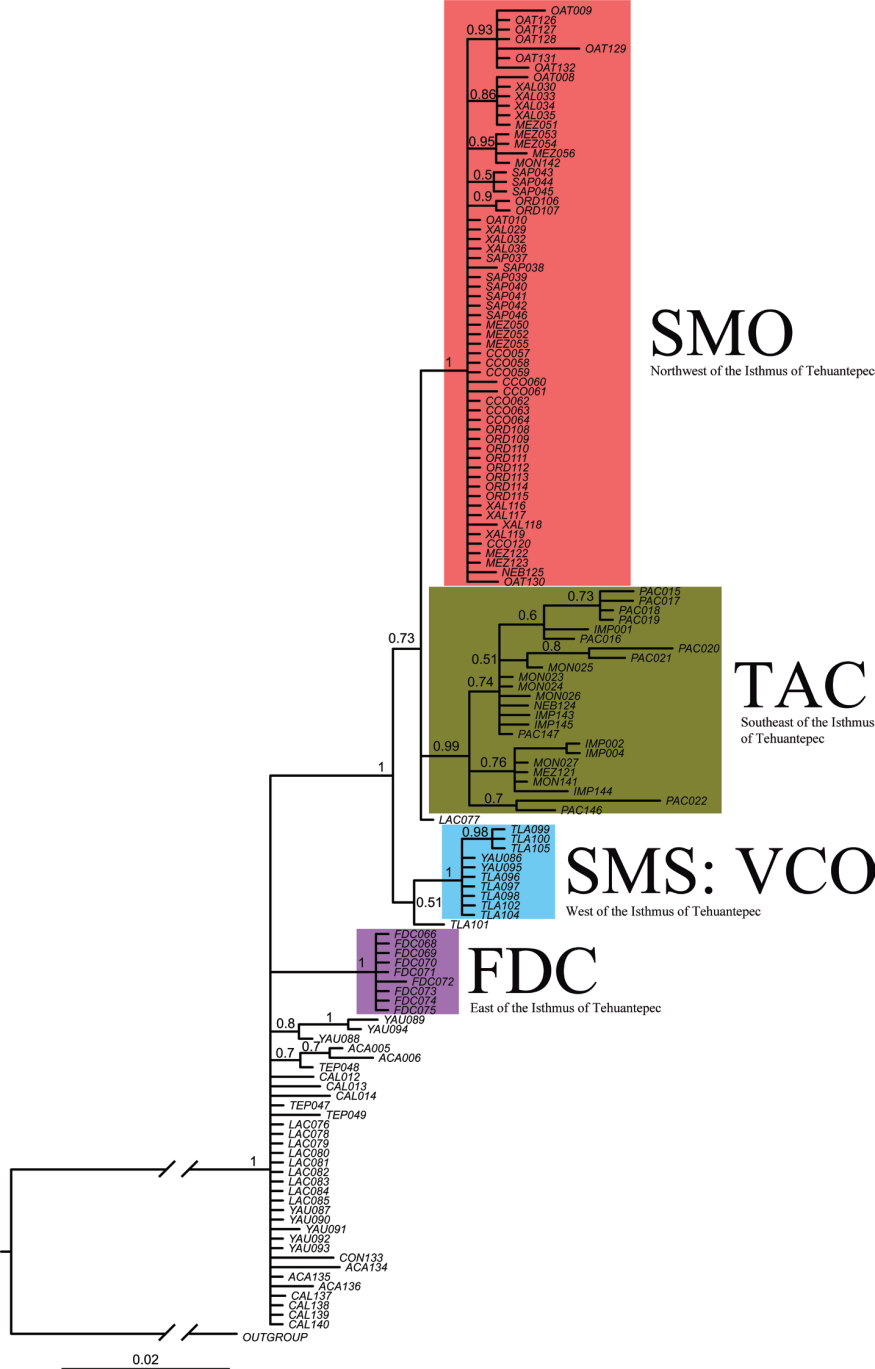
Genealogy recovered based on Bayesian inference. Numbers indicate the Bayesian posterior probabilities. The four main lineages are Flor de Chiapas (FDC), Sierra Madre del Sur that encompasses the Valles Centrales de Oaxaca (SMS: VCO), Tierras Altas de Chiapas (TAC), and Sierra Madre Oriental (SMO).

The best-fitting nucleotide substitution model was TrN+G+I. The model’s specific parameters were: base frequency = 0.3175, 0.1536 0.1489, 0.38; nst = 2; ts:tv ratio = 4.7931; Gamma shape = 0.5600; ncat = 4; and pinvar = 0.7490. The trees recovered by Bayesian (Fig. 3) and ML (See Suppl. material 4) inferences have congruent topologies, with slight changes in the internal relationships. The base of the *F.mexicana* clade is a polytomy formed by individuals of populations mainly distributed in the Sierra Madre del Sur, Pacific and Trans-Mexican Volcanic Belt, a Flor de Chiapas clade (FDC [PP = 1]), and a “big clade” (PP = 1) that encompasses the populations from Tierras Altas de Chiapas (TAC) to Sierra Madre Oriental (SMO). This “big clade” presents a substructure recovering a monophyletic group formed by Valles Centrales de Oaxaca (SMS: VCO) which is the sister group to the clade containing the rest of the sampled populations.

**Figure 4.**
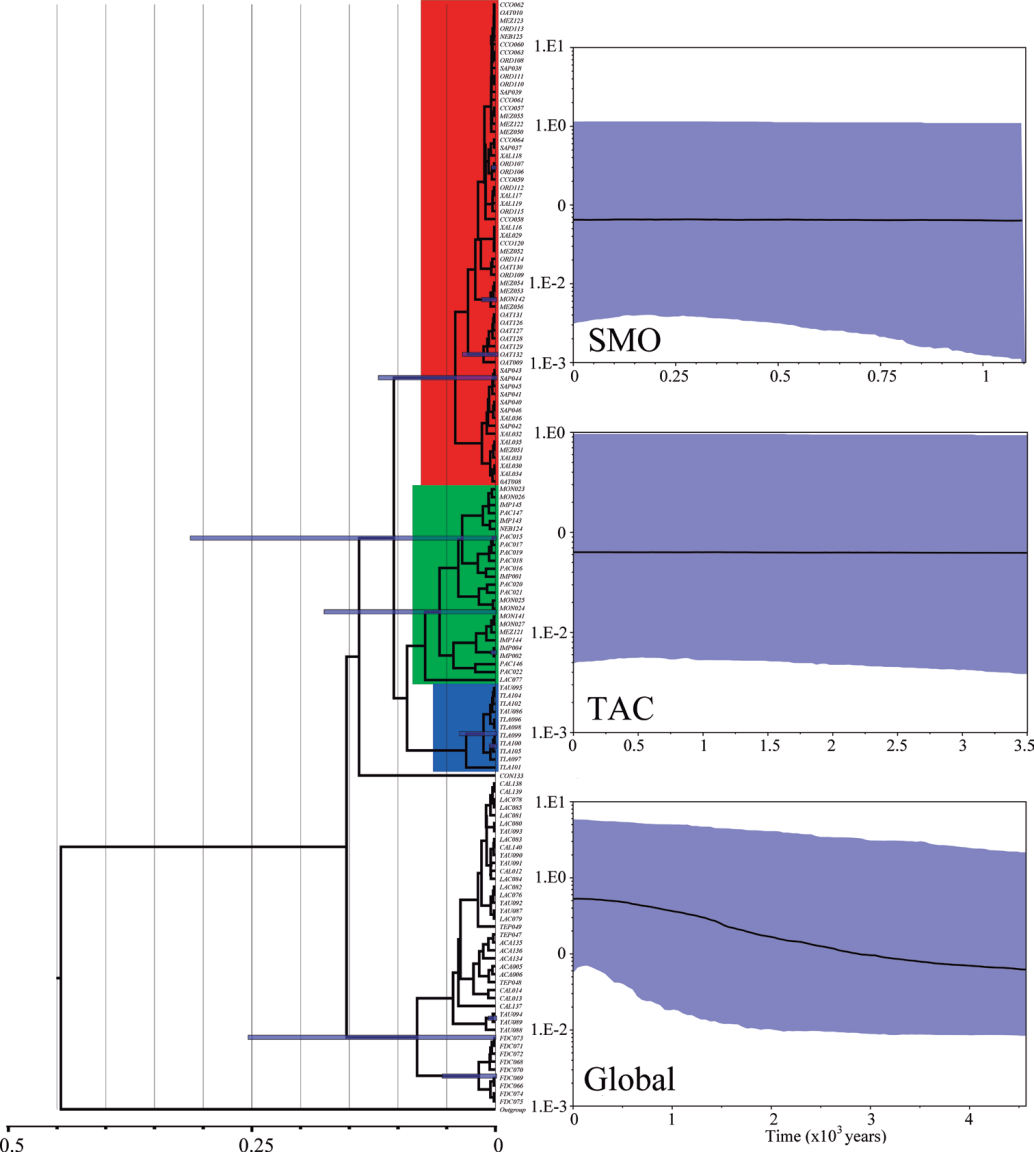
Chronogram and Skyline-plots based on COI sequences of *Falagoniamexicana*. For the chronogram the time is in millions of years. The Skyline-plots are shown for the clades going through demographic changes (SMO: Sierra Madre Oriental, TAC: Tierras Altas de Chiapas, and Global: Whole genealogy). Skyline-plots show mean *Ne*, plus 95% HDP confidence limit.

The chronogram (Fig. 4) showed a radiation event during the Middle Pleistocene. The results showed that *F.mexicana* split from the outgroup approximately 450,000 years ago. The diversification events that originated the main lineages within the species occurred nearly 150,000 years ago. The skyline-plots showed that the populations were kept in stasis for the most part of the Pleistocene, followed by a demographic expansion period towards the end of this epoch. For the lineage of TAC, a small increase in *Ne* occurred 3,500 years ago. In the SMO lineage, the increase of *Ne* occurred approximately 1,000 years ago. For the species (global analysis) the skyline-plot showed a demographic increment process that started approximately 4,000 years ago (Fig. 4).

## ﻿Discussion

### ﻿Population structure and genetic diversity

*Falagoniamexicana* is a highly polymorphic species with sixty different haplotypes recovered. Nucleotide changes are dispersed geographically creating many endemic haplotypes, but despite this, there are two widely distributed haplotypes (i.e., haplotypes H1 and H46) suggesting effective gene flow. Possibly, these haplotypes are ancestral that gave rise to the more recent ones. Gene flow is supported by the values obtained for migrants per generation (Table 5). Nonetheless, the low values of migrants per generation between the Sierra Madre Oriental (excluding Neblinas) and Tierras Altas de Chiapas (*M* = 0.113–0.643) suggest the existence of potential geographic barriers. The Isthmus of Tehuantepec could be such barrier. The gene flow is a complex process as suggested by the Mantel´s test. The mutation pattern observed in haplotypes H24 and H19 suggests that they might mediate the inferred gene flow. They are intermediate haplotypes between those from Sierra Madre Oriental, Tierras Altas de Chiapas, Tlalixtac, and Yautepec. There is a negative relationship between *Hd* and sample size (Table 2). High *Hd* values were obtained for populations of Tierras Altas de Chiapas, Ocampo-Acahuales-Tula, and Yautepec. Alternatively, populations closest to the Isthmus of Tehuantepec registered low *Hd* values. This occurs in the west (Lachiguiri) as well as in the east (Flor de Chiapas) of the Isthmus. However, the number of segregated sites and nucleotide diversity differ greatly within these two populations. The population of Lachiguiri has maintained gene flow with the Candelaria Loxicha and Yautepec populations (*M* = 9.323–inf; Table 5). Regarding to the population of Flor de Chiapas, it may have recently gone through a bottleneck reducing the genetic variability to a single segregated site. Such uneven genetic dynamic suggests that the Isthmus of Tehuantepec represents a geographic barrier that promoted differential effects on gene flow and demography.

The *F*_ST_ values (Table 5) and the AMOVA (Table 4) revealed a significant differentiation between most populations and a high genetic structure. We found a phylogeographic structure like that reported by Nolasco-Soto et al. (2017) for populations of the *Canthoncyanellus* beetle. According to these authors that species arose in the late Pliocene with radiation during the Pleistocene. The oldest lineages were recovered to the north of the Trans-Mexican Volcanic Belt (north of Sierra Madre Oriental) and in the Pacific, and the most recent lineage to the south of the Trans-Mexican Volcanic Belt, on the Coastal Plain of the Gulf of Mexico. Nolasco-Soto et al. (2017) reported limited historical gene flow with current genetic structure determined by isolation by distance and other contemporary processes. Anducho-Reyes et al. (2008) reported high *F*_ST_ for the bark beetle *Dendroctonusmexicanus*, reporting an allopatric fragmentation event among the populations to the north of the Trans-Mexican Volcanic Belt, and the Sierra Madre del Sur. They assumed a long-distance colonization process with continuous migration between populations of those geographic provinces. Another study by Sánchez-Sánchez et al. (2012) for the *Dendroctonusapproximatus* beetle found differentiation between populations in the group of Sierra Madre Oriental-Sierra Madre del Sur with respect to the Sierra Madre Occidental-Trans-Mexican Volcanic Belt group. This divergence was estimated to have occurred 195,000 years ago in the late Pleistocene. They concluded that the populations of that species represent a model of geographic range expansion and contraction, with subsequent restricted gene flow among putative Pleistocene refuges. Apparently, a rapid genetic structure in space and time for Coleoptera, and possibly for insects in general, is feasible in the mountainous regions of Mexico. It is important to continue with studies like these that will allow us to reinforce possible dominant evolutionary patterns in Neotropical insects.

### ﻿Biogeography and diversification time

The biogeographic history of Middle America has been complex and intriguing. One of the main questions has been how many events of exchange of organisms between the Nearctic and Neotropical regions have happened. Recently, studies have focused on the Mexican and Central America highlands to understand the biogeographic history of this area (Mastretta-Yanes et al. 2015). The estimated divergence time suggests that the origin of *F.mexicana* occurred during the Middle Pliocene, followed by a radiation that begun in the Upper Pleistocene that persisted during the Holocene. Such time coincide with several of the glacial-interglacial cycles that took place during those periods (O’Dea et al. 2016). Although most of the volcanic activity in the Sierra Madre Occidental and the other great mountain chains of Mexico ceased between the Oligocene-Miocene boundary; however, many localized and significant tectonic events have occurred until present times (de Cserna 1989; Ferrari et al. 1999, 2005, 2012). For instance, different pulses have conformed the topology of the Trans-Mexican Volcanic Belt were some of the large stratovolcanoes were formed less than 1 Mya. It has been found that the Trans-Mexican Volcanic Belt constitutes a geographical barrier, separating two large biogeographical assemblages, one to the north and the other to the south of it. Our results suggest that the ancestral area of *F.mexicana* might be located between the Pacific, Trans-Mexican Volcanic Belt or Sierra Madre del Sur biogeographic regions (Fig. 4). One of the northernmost records of the species is Sierra Alamos, Sonora (Santiago-Jiménez 2010). This area is located on the border of the Sierra Madre Occidental and Pacific provinces. That population is geographically closer to that of La Concordia, Sinaloa, representing a cluster of Pacific affinities; whereas the taxa distributed in the southern portion of the Sierra Madre Occidental have a closer affinity to those in the Sierra Madre del Sur (Marshall and Liebherr 2000).

The Isthmus of Tehuantepec is a geographical barrier for gene flow between the east and west regions. Many vicariance events have been invoked for different lineages (Castoe et al. 2009; Barber and Klicka 2010; Daza et al. 2010; González et al. 2011; Gutiérrez-Rodríguez et al. 2011). The Isthmus of Tehuantepec is a valley, located near the triple junction of the North American, Cocos and Caribbean plates. It is approximately 200 km long and has an average altitude of 200 m a.s.l. Climatic and topographic conditions along with the glacial-interglacial cycles during the Pleistocene might explaine diversification patterns found at this area (Pringle et al. 2012; Ornelas et al. 2013; Rodríguez-Gómez et al. 2013; Pech-May et al. 2019). Our findings are consistent with such vicariance patterns. Approximately 100,000 years ago three main clades of *Falagonia* rose: a) a cladogenetic event occurred separating the FDC clade (east of the Isthmus of Tehuantepec) from its sister group, b) the SMS: VCO clade (west of the Isthmus) differentiated from the TAC clade, and c) the SMO clade (northwest of the Isthmus) split from the TAC- SMS: VCO clade. All this could be regulated by continental uplift and sea level oscillations, or glacial-interglacial cycles.

The Isthmus of Tehuantepec has an average altitude of 200 m a.s.l. Although this is within the low range of the altitudinal tolerance of *Falagonia*, the dry and harsh climatic conditions of this area might create a barrier for this species. The northern area of the Isthmus is more humid due to the influence of the Gulf of Mexico. Until a century ago the predominant vegetation was tropical evergreen forest. In the past this vegetation was a continuum ecosystem from northern Veracruz to the northeast of Chiapas (Rzedowski 1994). However, anthropocentric activities (e.g., cattle ranching) have decreased the vegetation cover to near disappearance, altering the climatic conditions significantly. Such habitat modification indubitably is playing an important role in gene flow disruption among the populations of this staphylinid and many other species of flora and fauna. A more exhaustive population sampling at both sides of the Isthmus of Tehuantepec is necessary to understand the effects of deforestation and habitat fragmentation on *F.mexicana*.

For the SMO clade the Trans-Mexican Volcanic Belt constitutes a recent geographical barrier. One of the most relevant episodes occurred between the late Pliocene and the Quaternary in southern Veracruz (Palma Sola, Los Tuxtlas). Rodríguez et al. (2010) considered the Cofre de Perote-Pico de Orizaba volcanic range as the eastern end of the Trans-Mexican Volcanic Belt. The recent volcanic activity at this region and at the Sierra Madre Oriental allowed the SMO clade to finally establish during the Pleistocene, once geological, geographic, and climatic conditions were more stable. More recently, the volcanic activity to the east, at Chiconquiaco-Palma Sola, during the Quaternary, apparently constituted another barrier to the *F.mexicana* populations from the northeast of Mexico. The rocks of the Xalapa Monogenetic Volcanic Field, which comprises more than 59 volcanoes of the Quaternary, fall into three groups, the oldest with a little more than 2 Mya, others between 250 to 400 Kya, and others less than 100 Kya (Rodríguez et al. 2010). This recent geologic activity possibly has not allowed the differentiation of populations to the north (e.g., Ocampo-Acahuales-Tula) and south (e.g., Xalapa-La Orduña-San Antonio Paso del Toro) of Chiconquiaco-Palma Sola region.

The divergence times suggest an origin for the populations of *F.mexicana* in the western region of Mexico, where biogeographical assemblages from the southern Sierra Madre Occidental have been found more closely related to other assemblages to the south of the Trans-Mexican Volcanic Belt (Marshall and Liebherr 2000). Evidence of lineage diversification during the Pleistocene in Mexico and Central America has been found for different insects: dung beetles (Nolasco-Soto et al. 2017), bark beetles (Sánchez-Sánchez et al. 2012), edible crickets (Pedraza-Lara et al. 2015), dolichoderine ants (Pringle et al. 2012), and the *Triatomadimidiata* vector (Pech-May et al. 2019), among others. This supports the hypothesis that the geo-climatic events that took place during the Pliocene, such as intense volcanic activity, glacial-interglacial cycles, and sea level fluctuations, represent a major driver for the diversification of different insects’ lineages.

### ﻿Historical demography

The population expansion processes inferred for *F.mexicana* during the Quaternary period might be the result of the end of the last glacial maximum. The climate on Earth has undergone very marked fluctuations in cycles of ≈ 100,000 years, during the last 400,000 years. The coldest stages (8 °C less on average) constitute the glacial cycles, while the stages where the climates are equal to or warmer (2–3 °C higher) than the present, are the interglacials (Stute et al. 1995; Brown and Lomolino 1998). Glacial events in the Trans-Mexican Volcanic Belt produced a decrease in the Equilibrium Line Altitude of ~ 1,000 m from current levels in the main mountains. Therefore, not only the volcanic activity has contributed to the diversification of lineages in this area (Caballero et al. 2010). Such drastic changes in temperature also could have had an effect in fluctuations of the gene flow at the Isthmus of Tehuantepec region. Nowadays the lowlands that separate the higher altitude areas have arid zones with warmer climates, dry jungles, and tropical rain forests (Challenger and Soberón 2008; Mastretta-Yanes et al. 2015). During the last maximum glacial the paleolimnological and pollen data support drier and cooler conditions in central and eastern-central Mexico, particularly between 21,000 to 18,000 years ago. While deglaciation started earlier in temperate zones, conditions remained very similar to the last maximum glacial for the Trans-Mexican Volcanic Belt mountains (Caballero et al. 2010). *Falagoniamexicana* could have been distributed in a northwest-southeast sequence reaching northern Central America, pushed by the effects of the Wisconsin glaciation and other previous glaciations, while a displacement in the opposite direction (i.e., invasion of the Sierra Madre Oriental) occurred more recently. Other data obtained from the mtDNA from a variety of insect groups has revealed a general pattern where population expansions occurred during the Pleistocene (Pfeiler and Markow 2011; Pfeiler et al. 2013; Nolasco-Soto et al. 2017; Pech-May et al. 2019).

### ﻿Association with ants

The larvae of *F.mexicana* are unknown; therefore, there is a lack of information about its natural history, behavior, and ecology. So far, the adults of this species have been only observed and collected in association or near by the nests of *Attamexicana*. Santiago-Jiménez (2010) reports that the adults are active predator of these ants. However, alternative hypotheses exist to explain the association between *F.mexicana* with *A.mexicana*. The ant species of the tribe Attini depend strictly for their feeding on the cultivation of the *Leucoagaricusgongylophorus* fungi. Such agricultural practice creates particular micro environmental condition inside the ant´s nest as well as the continuous production of foliar debris. Temperature fluctuations had a harsh effect on insect population perhaps partly due to thermoregulation in conjunction with body mass, playing an important role at the level of ecological niche and geographic distribution patterns. Verdú et al. (2006) pointed out that dung beetles weighing less than 1.9 g, tend to be thermo-conformal or poikilotherms, only capable of maintaining a temperature similar to the environment at the time of flight. Márquez and Navarrete-Heredia (1994) reported that the debris maintain, most of the time, a temperature between 19–27 °C, usually higher than environmental temperature. This suggests that *F.mexicana* can use the nests of *A.mexicana* as a refuge to regulate their body temperature and, at the same time, be able to capture prey. Therefore, the external debris of *A.mexicana* may historically constitute a refuge without abrupt temperature changes, allowing this species to occupy higher altitudinal and wider latitudinal ranges in different vegetation types.

### ﻿Final remarks

*Falagoniamexicana* is a species whose origin can be dated during the Pleistocene and diversified in at least four main haplogroups since then. Geographic barriers such as the Isthmus of Tehuantepec, Trans-Mexican Volcanic Belt and Sierra Madre Oriental have played a major role in the evolution of this species. Even though geographic regions define the haplogroups, evidence of contemporary restricted gene flow was found, which indicates that this lineage is in the process of genetic differentiation. The differentiation among populations was supported by significant values of pairwise *F*_st_, and the global genetic structure revealed by the AMOVA. Also, a significantly positive correlation between genetic and geographic distances, suggesting isolation by distance is a process promoting its genetic structure. Finally, climatic, and volcanic events that occurred during the late Quaternary Period indubitably have shaped the distribution, genetic structure, and demography history of this elusive insect.

## Supplementary materials

Supplementary material 1 Chronogram and Skyline-plots based on COI sequences of *Falagoniamexicana* Data type chronogram Explanation note For the chronogram the time is in millions of years. The Skyline-plots are shown for the clades going through demographic changes (i.e., Sierra Madre Oriental, Tierras Altas de Chiapas, and global genealogy). On each Skyline-plot, the red line shows the trend of the mean Ne, and blue lines represent 95% HDP confidence limit. Substitution rate of 2.0 (10-2 subs/site/My/lineage) was used. https://binary.pensoft.net/file/831203This dataset is made available under the Open Database License (http://opendatacommons.org/licenses/odbl/1.0/). The Open Database License (ODbL) is a license agreement intended to allow users to freely share, modify, and use this Dataset while maintaining this same freedom for others, provided that the original source and author(s) are credited.Justo A. Reyes, Alejandro Espinosa de los Monteros, Quiyari J. Santiago-Jiménez

Supplementary material 2 Variable sites of the COI nucleotide sequences of *F.mexicana* Data type nucleotides Explanation note Variable sites of the COI nucleotide sequences from *F.mexicana*. https://binary.pensoft.net/file/831204This dataset is made available under the Open Database License (http://opendatacommons.org/licenses/odbl/1.0/). The Open Database License (ODbL) is a license agreement intended to allow users to freely share, modify, and use this Dataset while maintaining this same freedom for others, provided that the original source and author(s) are credited.Justo A. Reyes, Alejandro Espinosa de los Monteros, Quiyari J. Santiago-Jiménez

Supplementary material 3 Table of haplotype frequencies found in COI sequences of *F.mexicana* by population Data type haplotype frequencies Explanation note Haplotype frequencies found in the 472 bp fragment of COI sequences from *F.mexicana* by population. https://binary.pensoft.net/file/831205This dataset is made available under the Open Database License (http://opendatacommons.org/licenses/odbl/1.0/). The Open Database License (ODbL) is a license agreement intended to allow users to freely share, modify, and use this Dataset while maintaining this same freedom for others, provided that the original source and author(s) are credited.Justo A. Reyes, Alejandro Espinosa de los Monteros, Quiyari J. Santiago-Jiménez

Supplementary material 4 Maximum likelihood tree recovered from sequences of the 472 bp fragment of COI from *Falagoniamexicana* Data type phylogenetic tree Explanation note Ln likelihood= -1606.43. The four main lineages identified are Flor de Chiapas (FDC), Sierra Madre del Sur that encompasses the Valles Centrales de Oaxaca (SMS: VCO), Tierras Altas de Chiapas (TAC), and Sierra Madre Oriental (SMO). https://binary.pensoft.net/file/831206This dataset is made available under the Open Database License (http://opendatacommons.org/licenses/odbl/1.0/). The Open Database License (ODbL) is a license agreement intended to allow users to freely share, modify, and use this Dataset while maintaining this same freedom for others, provided that the original source and author(s) are credited.Justo A. Reyes, Alejandro Espinosa de los Monteros, Quiyari J. Santiago-Jiménez

Supplementary material 5 Matrix Data type matrix Explanation note Matrix used in the analyses. https://binary.pensoft.net/file/831207This dataset is made available under the Open Database License (http://opendatacommons.org/licenses/odbl/1.0/). The Open Database License (ODbL) is a license agreement intended to allow users to freely share, modify, and use this Dataset while maintaining this same freedom for others, provided that the original source and author(s) are credited.Justo A. Reyes, Alejandro Espinosa de los Monteros, Quiyari J. Santiago-Jiménez
