# Supplementary material for: ﻿Phylogeography of Falagoniamexicana Sharp, 1883 (Coleoptera, Staphylinidae, Aleocharinae)
Source: Zookeys. 2023 Mar 29;1156:107–31. doi: 10.3897/zookeys.1156.84943 (PMC10209309; doi:10.3897/zookeys.1156.84943)
Supplement: Supplementary material 1 — Chronogram and Skyline-plots based on COI sequences of Falagoniamexicana [file zookeys-1156-107_article-84943__-s001.pdf]

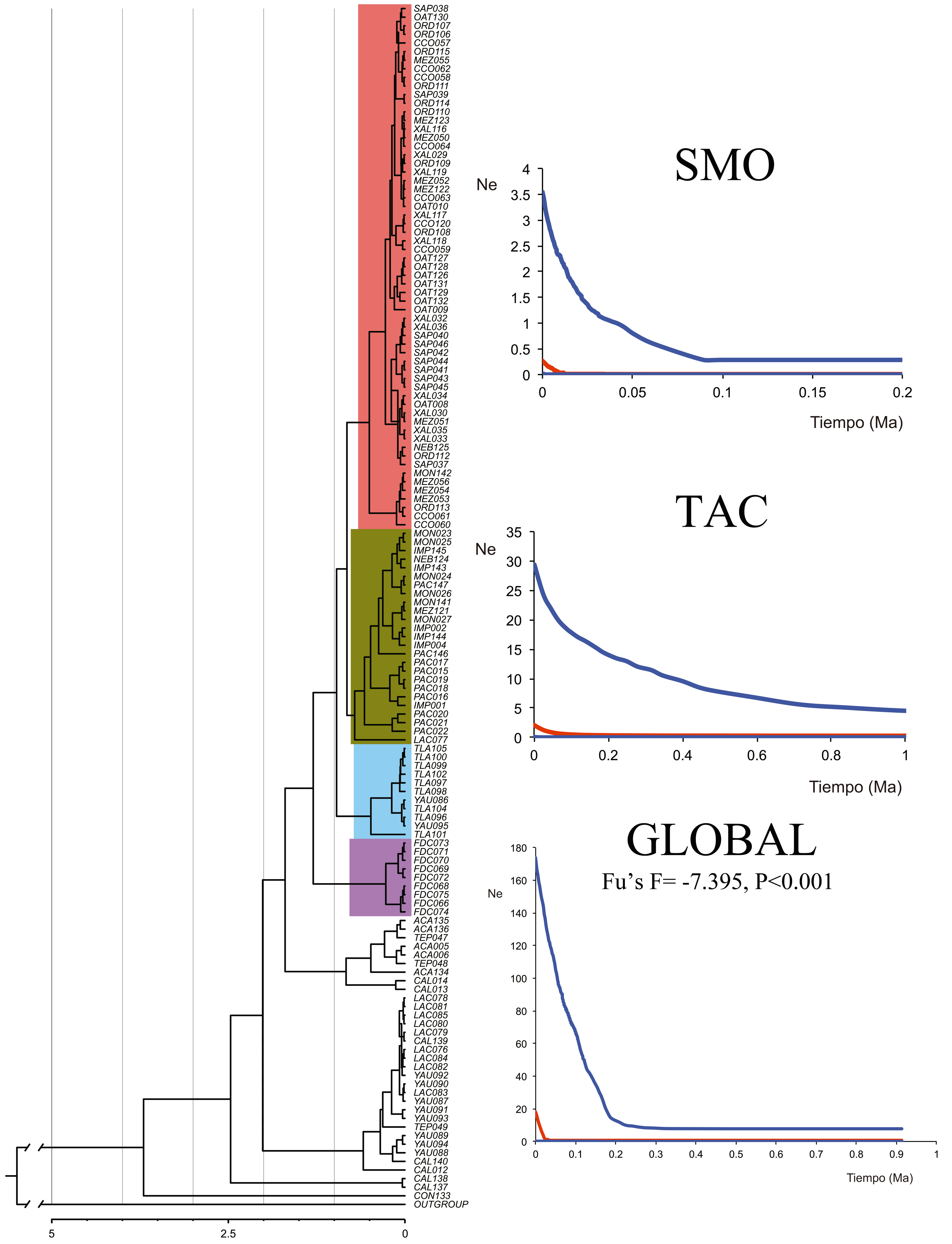

Supplementary file 1: Figure 1. Chronogram and Skyline-plots based on COI sequences of *Falagonia mexicana*. For the chronogram the time is in millions of years. The Skyline-plots are shown for the clades going through demographic changes (i.e., Sierra Madre Oriental, Tierras Altas de Chiapas, and global genealogy). On each Skyline-plot, the red line shows the trend of the mean *Ne*, and blue lines represent 95% HDP confidence limit. A substitution rate of 2.0 (10^{-2}subs/site/My/lineage) was used.
