# Supplementary material for: ﻿Phylogeography of Falagoniamexicana Sharp, 1883 (Coleoptera, Staphylinidae, Aleocharinae)
Source: Zookeys. 2023 Mar 29;1156:107–31. doi: 10.3897/zookeys.1156.84943 (PMC10209309; doi:10.3897/zookeys.1156.84943)
Supplement: Supplementary material 3 — Table of haplotype frequencies found in COI sequences of F.mexicana by population [file zookeys-1156-107_article-84943__-s003.pdf]

Supplementary file 3: Table 2. Table of haplotype frequencies found in COI sequences of *F. mexicana* by population

	H1	H2	H3	H4	H5	H6	H7	H8	H9	H10	H11	H12	H13	H14	H15	H16	H17	H18	H19	H20	H21	H22	H23	H24	H25	H26	H27	H28	H29	H30
Montecristo	0	0	0	0	0	1	0	0	0	0	0	0	0	0	0	0	0	0	0	2	0	0	2	0	0	1	0	0	0	1
El Imposible	0	0	0	0	0	0	0	0	0	0	0	0	0	0	0	0	0	0	0	0	1	0	0	0	1	0	1	1	0	0
Pacaya	0	0	0	0	0	0	0	0	0	0	0	0	0	0	0	0	0	0	0	0	0	1	1	0	0	0	0	0	1	0
Acahuizotla	0	0	0	0	0	0	0	0	0	0	0	0	0	0	0	0	0	0	0	0	0	0	0	0	0	0	0	0	0	0
San Agustin Mezquititlan	5	0	0	0	0	2	1	0	0	0	0	0	0	0	1	0	0	0	0	1	0	0	0	0	0	0	0	0	0	0
Candelaria Loxicha	0	0	0	0	0	0	0	0	0	0	0	0	0	0	0	0	0	0	0	0	0	0	0	0	0	0	0	0	0	0
Flor de Chiapas	0	0	0	0	0	0	0	0	0	0	0	0	0	0	0	0	0	0	0	0	0	0	0	0	0	0	0	0	0	0
Lachiguiri	0	0	0	0	0	0	0	0	0	0	0	0	0	0	0	0	0	0	1	0	0	0	0	0	0	0	0	0	0	0
Tlalixtac	0	0	0	0	0	0	0	0	0	0	0	0	0	0	0	0	0	0	0	0	0	0	0	0	0	0	0	0	0	0
Yautepec	0	0	0	0	0	0	0	0	0	0	0	0	0	0	0	0	0	0	0	0	0	0	0	0	0	0	0	0	0	0
Tepexco	0	0	0	0	0	0	0	0	0	0	0	0	0	0	0	0	0	0	0	0	0	0	0	0	0	0	0	0	0	0
Neblinas	0	1	0	0	0	0	0	0	0	0	0	0	0	0	0	0	0	0	0	0	0	0	0	1	0	0	0	0	0	0
La Concordia	0	0	0	0	0	0	0	0	0	0	0	0	0	0	0	0	0	0	0	0	0	0	0	0	0	0	0	0	0	0
Ocampo-Acahuales-Tula	1	0	0	1	0	0	0	0	0	0	4	1	1	1	0	1	0	0	0	0	0	0	0	0	0	0	0	0	0	0
Cerro Colorado	7	0	1	0	0	0	0	1	0	0	0	0	0	0	0	0	0	0	0	0	0	0	0	0	0	0	0	0	0	0
La Orduña	8	0	0	0	0	0	0	0	2	0	0	0	0	0	0	0	0	0	0	0	0	0	0	0	0	0	0	0	0	0
San Antonio Paso del Toro	2	0	0	0	0	0	0	0	0	1	0	0	0	0	0	0	4	3	0	0	0	0	0	0	0	0	0	0	0	0
Xalapa	4	0	0	0	1	0	0	0	0	0	0	0	0	0	4	0	2	0	0	0	0	0	0	0	0	0	0	0	0	0
**Frequency**	**27**	**1**	**1**	**1**	**1**	**3**	**1**	**1**	**2**	**1**	**4**	**1**	**1**	**1**	**5**	**1**	**6**	**3**	**1**	**3**	**1**	**1**	**3**	**1**	**1**	**1**	**1**	**1**	**1**	**1**

	H31	H32	H33	H34	H35	H36	H37	H38	H39	H40	H41	H42	H43	H44	H45	H46	H47	H48	H49	H50	H51	H52	H53	H54	H55	H56	H57	H58	H59	H60	Total
Montecristo	0	0	0	0	0	0	0	0	0	0	0	0	0	0	0	0	0	0	0	0	0	0	0	0	0	0	0	0	0	0	**7**
El Imposible	0	0	0	0	0	2	0	0	0	0	0	0	0	0	0	0	0	0	0	0	0	0	0	0	0	0	0	0	0	0	**6**
Pacaya	1	1	1	1	2	0	1	0	0	0	0	0	0	0	0	0	0	0	0	0	0	0	0	0	0	0	0	0	0	0	**10**
Acahuizotla	0	0	0	0	0	0	0	0	0	0	0	0	0	0	0	0	0	0	0	0	0	0	0	0	1	1	1	0	1	1	**5**
San Agustin Mezquititlan	0	0	0	0	0	0	0	0	0	0	0	0	0	0	0	0	0	0	0	0	0	0	0	0	0	0	0	0	0	0	**10**
Candelaria Loxicha	0	0	0	0	0	0	0	0	0	0	0	0	0	0	1	3	1	1	1	0	0	0	0	0	0	0	0	0	0	0	**7**
Flor de Chiapas	0	0	0	0	0	0	0	0	0	0	6	2	1	0	0	0	0	0	0	0	0	0	0	0	0	0	0	0	0	0	**9**
Lachiguiri	0	0	0	0	0	0	0	0	0	0	0	0	0	0	0	9	0	0	0	0	0	0	0	0	0	0	0	0	0	0	**10**
Tlalixtac	0	0	0	0	0	0	0	1	5	3	0	0	0	0	0	0	0	0	0	0	0	0	0	0	0	0	0	0	0	0	**9**
Yautepec	0	0	0	0	0	0	0	0	2	0	0	0	0	0	0	4	0	0	0	1	1	1	1	0	0	0	0	0	0	0	**10**
Tepexco	0	0	0	0	0	0	0	0	0	0	0	0	0	0	0	0	0	0	0	0	0	0	0	1	1	0	0	1	0	0	**3**
Neblinas	0	0	0	0	0	0	0	0	0	0	0	0	0	0	0	0	0	0	0	0	0	0	0	0	0	0	0	0	0	0	**2**
La Concordia	0	0	0	0	0	0	0	0	0	0	0	0	0	1	0	0	0	0	0	0	0	0	0	0	0	0	0	0	0	0	**1**
Ocampo-Acahuales-Tula	0	0	0	0	0	0	0	0	0	0	0	0	0	0	0	0	0	0	0	0	0	0	0	0	0	0	0	0	0	0	**10**
Cerro Colorado	0	0	0	0	0	0	0	0	0	0	0	0	0	0	0	0	0	0	0	0	0	0	0	0	0	0	0	0	0	0	**9**
La Orduña	0	0	0	0	0	0	0	0	0	0	0	0	0	0	0	0	0	0	0	0	0	0	0	0	0	0	0	0	0	0	**10**
San Antonio Paso del Toro	0	0	0	0	0	0	0	0	0	0	0	0	0	0	0	0	0	0	0	0	0	0	0	0	0	0	0	0	0	0	**10**
Xalapa	0	0	0	0	0	0	0	0	0	0	0	0	0	0	0	0	0	0	0	0	0	0	0	0	0	0	0	0	0	0	**11**
**Frequency**	**1**	**1**	**1**	**1**	**2**	**2**	**1**	**1**	**7**	**3**	**6**	**2**	**1**	**1**	**1**	**16**	**1**	**1**	**1**	**1**	**1**	**1**	**1**	**1**	**2**	**1**	**1**	**1**	**1**	**1**	**139**
