# Supplementary material for: ﻿Phylogeography of Falagoniamexicana Sharp, 1883 (Coleoptera, Staphylinidae, Aleocharinae)
Source: Zookeys. 2023 Mar 29;1156:107–31. doi: 10.3897/zookeys.1156.84943 (PMC10209309; doi:10.3897/zookeys.1156.84943)
Supplement: Supplementary material 4 — Maximum likelihood tree recovered from sequences of the 472 bp fragment of COI from Falagoniamexicana [file zookeys-1156-107_article-84943__-s004.pdf]

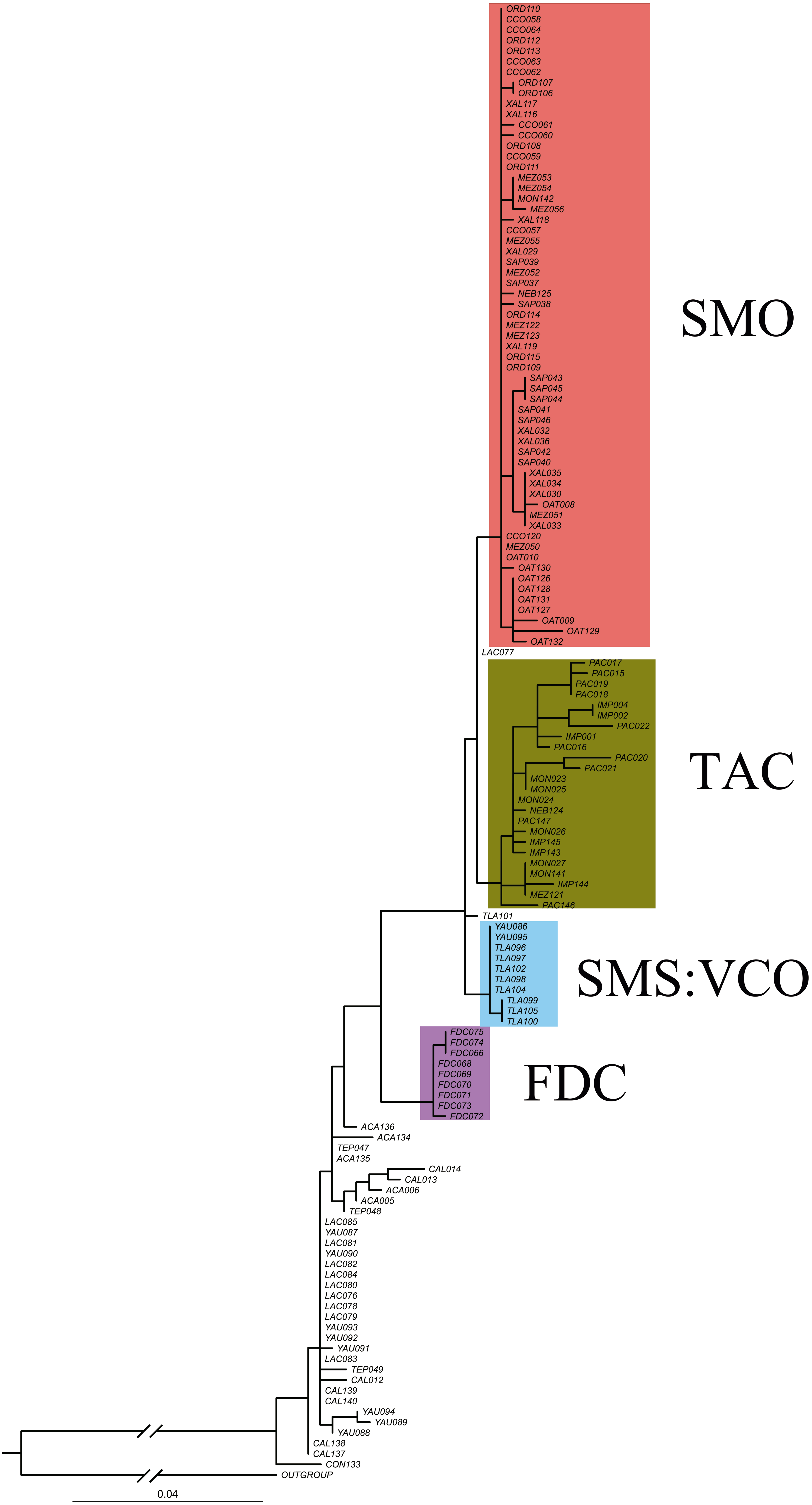

Supplementary file 4: Figure 2. Maximum likelihood tree recovered from sequences of the 472 bp fragment of CO1 from *Falagonia mexicana*. Ln Likelihood= -1606.43. The four main lineages identified are Flor de Chiapas (FDC), Sierra Madre del Sur that encompasses the Valles Centrales de Oaxaca (SMS: VCO), Tierras Altas de Chiapas (TAC), and Sierra Madre Oriental (SMO).
