# Supplementary material for: ﻿Phylogeography of Falagoniamexicana Sharp, 1883 (Coleoptera, Staphylinidae, Aleocharinae)
Source: Zookeys. 2023 Mar 29;1156:107–31. doi: 10.3897/zookeys.1156.84943 (PMC10209309; doi:10.3897/zookeys.1156.84943)
Supplement: Supplementary material 2 — Variable sites of the COI nucleotide sequences of F.mexicana [file zookeys-1156-107_article-84943__-s002.pdf]

Supplementary file 2: Table 1. Variable sites of the COI nucleotide sequences of *F. mexicana*

	10	13	16	19	23	27	31	34	37	40	46	49	50	61	62	64	68	70	82	87	89	112	115	133	136	142	163	169	175	178	196	206	224	244	247	262	304	328	329	331	332	334	343	397	412	418	421	428	430	442	445	449	458	466	468
Haplotype 1	?	?	?	?	A	C	T	A	A	A	T	A	A	G	T	A	C	C	T	T	A	A	A	A	C	A	T	A	C	C	A	G	T	T	T	A	A	A	T	A	T	A	C	T	T	A	A	G	T	C	A	C	T	A	A
Haplotype 2	T	G	T	A	A	C	T	A	A	A	T	A	A	A	T	A	C	C	T	T	A	A	A	A	C	A	T	A	C	C	A	G	T	T	T	A	A	A	T	A	T	A	C	T	T	A	A	G	T	C	A	C	T	A	A
Haplotype 3	T	G	T	A	A	C	T	A	A	A	T	A	A	G	T	A	C	C	T	T	A	A	G	A	C	A	T	A	C	C	A	G	T	T	T	A	A	A	T	A	T	A	C	T	T	A	A	G	T	C	A	C	T	A	A
Haplotype 4	T	G	T	A	A	C	T	A	A	A	T	A	A	G	T	A	C	C	T	T	A	A	A	A	C	A	T	T	C	C	A	G	T	T	T	A	A	A	T	A	T	A	C	T	T	A	A	G	T	C	A	C	T	A	A
Haplotype 5	T	G	T	A	A	C	T	A	A	A	T	A	A	G	T	A	A	C	T	T	A	A	A	A	C	A	T	A	C	C	A	G	T	T	T	A	A	A	T	A	T	A	C	T	T	A	A	G	T	C	A	C	T	A	A
Haplotype 6	T	G	T	A	A	C	T	A	A	A	T	A	A	G	T	A	C	C	T	T	A	A	A	A	C	A	T	A	C	C	A	G	C	T	T	A	A	A	T	A	T	A	C	T	T	A	A	G	T	C	A	C	T	A	A
Haplotype 7	?	?	T	A	A	C	T	A	A	A	T	A	A	G	T	A	C	C	T	T	A	A	A	A	C	A	T	G	C	C	A	G	C	T	T	A	A	A	T	A	T	A	C	T	T	A	A	G	T	C	A	C	T	A	A
Haplotype 8	T	G	T	A	A	C	T	A	A	A	T	A	A	G	T	A	C	C	T	T	A	A	A	A	C	A	T	A	C	C	G	G	T	T	T	A	A	A	T	A	T	A	C	T	T	A	A	G	T	C	A	C	T	?	?
Haplotype 9	T	G	T	A	A	C	T	A	A	A	T	A	A	G	T	A	C	C	T	T	A	A	A	A	C	A	T	A	C	C	A	G	T	T	T	A	A	A	T	A	T	A	C	T	T	A	A	G	T	C	A	T	T	A	A
Haplotype 10	T	G	T	A	A	C	T	G	A	A	T	A	A	G	T	A	C	C	T	T	A	A	A	A	C	A	T	A	C	C	A	G	T	T	T	A	A	A	T	A	T	A	C	T	T	A	A	G	T	C	A	C	T	A	A
Haplotype 11	T	G	T	A	A	C	T	A	A	A	T	A	A	G	T	G	C	C	T	T	A	A	A	A	C	A	T	A	C	C	A	G	T	T	T	A	A	A	T	A	T	A	C	T	T	A	A	G	T	C	A	C	T	A	A
Haplotype 12	A	G	T	A	A	C	T	A	A	G	T	A	A	G	T	G	C	C	T	T	A	A	A	A	C	A	T	A	C	C	A	G	T	T	T	A	A	A	T	A	T	A	C	T	T	A	A	T	T	C	A	C	G	A	A
Haplotype 13	T	G	T	A	A	C	T	A	A	A	T	A	A	G	T	G	C	C	T	T	A	A	A	A	C	A	T	A	C	C	A	A	T	T	T	A	A	A	T	A	T	A	C	T	T	A	A	G	T	?	?	?	?	?	?
Haplotype 14	T	G	T	A	A	C	A	A	G	A	T	A	A	G	T	G	C	C	T	T	A	A	A	A	C	A	T	A	C	C	A	G	T	T	T	A	A	A	T	A	T	A	C	T	T	A	A	G	T	C	A	C	T	A	A
Haplotype 15	T	G	T	G	A	C	T	A	G	A	T	A	A	G	T	A	C	C	T	T	A	A	A	A	C	A	T	A	C	C	A	G	T	T	T	A	A	A	T	A	T	A	C	T	T	A	A	G	T	C	A	C	T	A	A
Haplotype 16	?	?	?	?	?	C	T	A	G	G	T	A	A	G	T	A	C	C	T	T	A	A	A	A	C	A	T	A	C	C	A	G	T	T	T	A	A	A	T	A	T	A	C	T	T	A	A	G	T	C	A	C	T	A	A
Haplotype 17	T	G	T	G	A	C	T	A	A	A	T	A	A	G	T	A	C	C	T	T	A	A	A	A	C	A	T	A	C	C	A	G	T	T	T	A	A	A	T	A	T	A	C	T	T	A	A	G	T	C	A	C	T	A	A
Haplotype 18	A	G	T	G	A	C	T	A	A	A	T	A	A	G	T	A	C	C	T	T	A	A	A	A	C	A	T	A	C	C	A	G	T	T	T	A	A	A	T	A	T	A	C	T	T	A	A	G	T	C	A	C	T	A	A
Haplotype 19	T	G	T	A	A	C	T	A	A	A	T	A	A	G	T	A	C	T	T	T	A	A	A	A	C	A	T	A	C	T	A	G	T	T	T	A	A	A	T	A	T	A	C	T	T	A	A	G	T	C	A	C	T	A	?
Haplotype 20	T	G	T	A	A	C	T	A	A	A	C	A	A	G	T	A	C	T	T	T	A	A	A	A	T	G	T	A	C	T	A	G	T	T	T	A	A	A	T	A	T	A	C	T	T	A	A	G	T	C	G	C	T	A	A
Haplotype 21	?	?	?	?	?	?	?	?	?	?	?	?	?	G	T	A	C	T	T	T	A	A	A	A	T	G	T	A	C	T	A	G	T	T	T	A	A	A	T	A	T	A	C	C	T	G	A	G	T	C	G	C	T	A	A
Haplotype 22	T	G	T	A	A	C	T	A	A	A	C	A	A	G	T	A	C	T	T	T	A	A	A	A	T	A	C	G	C	T	A	G	T	T	T	A	A	A	T	A	T	G	C	T	T	A	A	G	T	C	A	C	T	A	A
Haplotype 23	?	?	?	?	?	?	T	A	A	A	C	A	A	G	T	A	C	T	T	T	A	A	A	G	T	A	T	A	C	T	A	G	T	T	T	A	A	A	T	A	T	A	C	T	T	A	A	G	T	C	A	C	T	A	A
Haplotype 24	T	G	T	A	A	C	T	A	A	A	C	A	A	G	C	A	C	T	T	T	A	A	A	G	T	A	T	A	C	T	A	G	T	T	T	A	A	A	T	A	T	A	C	T	T	A	A	G	T	C	A	C	T	A	A
Haplotype 25	T	G	T	A	A	C	T	A	A	A	C	A	A	G	T	A	C	T	T	T	A	A	A	G	T	A	T	A	C	T	A	G	T	T	T	A	A	A	T	A	T	A	C	T	T	A	G	G	T	C	A	C	T	A	A
Haplotype 26	T	G	T	A	A	C	T	G	A	A	C	A	A	G	T	A	C	T	T	T	A	A	A	G	T	A	T	A	C	T	A	G	T	T	T	A	A	A	T	A	T	A	C	T	T	A	A	G	T	C	A	C	T	A	A
Haplotype 27	T	G	T	A	A	C	T	A	A	A	C	A	A	G	T	A	C	T	T	T	A	G	A	G	T	A	T	A	C	T	A	G	T	T	T	A	A	A	T	A	T	A	C	T	T	A	A	G	T	C	A	C	T	A	A
Haplotype 28	T	A	T	A	A	C	A	A	G	A	C	A	A	G	T	A	C	T	T	T	A	G	A	G	T	A	T	A	C	T	A	G	T	T	T	A	A	A	T	A	T	A	C	T	T	A	A	G	T	C	A	C	T	A	A
Haplotype 29	T	G	T	A	A	C	A	A	G	A	C	A	A	G	T	A	C	T	T	T	A	A	A	G	T	A	T	A	C	T	A	G	T	T	T	A	A	A	T	A	T	A	C	T	T	G	A	G	T	C	A	C	T	A	A
Haplotype 30	?	?	T	G	A	C	T	A	A	A	C	A	A	G	T	A	C	T	T	T	A	A	A	G	T	A	T	A	C	T	A	G	T	T	T	A	A	A	T	A	T	A	C	T	T	A	A	G	T	C	A	C	T	A	A
Haplotype 31	?	?	?	?	A	C	T	A	G	G	A	G	A	G	T	A	C	T	T	C	A	A	A	G	T	A	T	A	C	T	A	G	T	T	T	A	A	A	T	A	T	A	C	T	T	A	A	G	T	C	A	C	T	G	A
Haplotype 32	?	?	T	G	A	C	T	A	A	G	G	A	A	G	C	A	C	T	T	T	A	A	A	G	T	A	T	A	C	T	A	G	T	T	T	A	A	A	T	A	T	A	C	T	T	A	A	G	T	C	A	C	T	G	A
Haplotype 33	?	?	?	?	?	?	?	?	?	?	?	A	A	G	C	A	C	T	T	T	A	A	A	G	T	A	T	A	C	T	A	G	T	T	T	A	T	A	T	A	T	A	C	T	T	A	A	G	T	C	A	C	T	G	A
Haplotype 34	?	?	?	?	G	A	A	A	G	A	C	A	A	G	C	A	C	T	T	T	A	A	A	G	T	A	T	A	C	T	A	G	T	T	T	A	A	A	T	A	T	A	C	T	T	A	A	G	T	C	A	C	T	G	A
Haplotype 35	?	?	?	?	?	C	A	A	G	A	C	A	A	G	C	A	C	T	T	T	A	A	A	G	T	A	T	A	C	T	A	G	T	T	T	A	A	A	T	A	T	A	C	T	T	A	A	G	T	C	A	C	T	G	A
Haplotype 36	?	?	?	?	?	C	A	G	G	A	C	A	A	G	T	A	C	T	T	T	A	A	A	A	T	G	T	A	C	T	A	G	T	T	T	A	A	A	T	A	T	A	C	T	T	A	A	G	T	C	G	C	T	A	A
Haplotype 37	?	?	?	?	C	C	A	G	G	G	G	A	A	A	T	A	C	T	T	T	A	A	A	A	T	A	C	G	C	T	A	G	T	T	T	A	A	A	T	A	T	A	C	T	T	A	A	G	T	C	A	C	T	A	A
Haplotype 38	T	G	T	A	A	C	T	A	A	A	T	A	A	A	T	A	C	T	T	T	A	A	A	A	C	A	T	G	C	T	A	G	T	T	T	A	A	A	T	A	T	A	C	T	T	A	A	G	T	C	A	C	T	A	A
Haplotype 39	T	G	T	A	A	C	T	A	A	A	T	A	A	A	T	A	C	T	T	T	A	A	A	A	C	A	T	A	C	T	A	G	T	T	T	A	G	A	T	A	T	A	C	T	T	A	A	G	T	T	A	C	T	?	?
Haplotype 40	T	G	T	A	A	C	T	A	A	A	T	A	A	A	T	A	C	T	T	T	A	A	A	A	C	A	T	A	C	T	A	G	T	T	T	A	G	A	T	A	T	A	C	T	T	A	A	G	T	T	A	T	T	A	A
Haplotype 41	A	G	T	G	A	C	T	A	A	A	T	A	A	A	T	A	C	T	C	T	A	A	T	T	C	A	C	A	C	T	A	G	T	T	T	G	A	A	T	G	C	A	T	T	T	A	A	G	T	C	A	T	?	?	?
Haplotype 42	A	G	T	G	A	C	T	A	A	A	T	A	A	A	T	A	C	T	C	T	A	A	T	T	C	A	C	A	C	T	A	G	T	T	T	G	A	A	T	G	C	A	T	T	T	A	A	G	T	C	A	C	T	A	?
Haplotype 43	A	G	T	G	A	C	T	A	A	A	T	A	A	A	T	A	C	T	C	T	A	A	T	T	C	A	C	A	C	T	A	G	T	T	T	G	A	A	T	G	C	A	T	T	T	A	A	G	T	T	A	C	T	A	?
Haplotype 44	T	G	G	A	A	C	T	A	A	A	T	A	A	G	T	A	C	T	T	T	A	A	T	A	C	A	C	A	T	T	A	G	T	C	T	A	A	A	T	A	C	A	T	T	C	A	A	G	T	C	A	T	T	A	A
Haplotype 45	T	G	T	G	A	C	T	A	A	A	T	A	A	A	T	A	C	T	T	T	A	A	T	T	C	A	C	A	C	T	A	G	T	C	T	A	A	A	C	A	C	A	T	T	C	A	A	G	T	C	A	T	T	A	A
Haplotype 46	T	G	T	G	A	C	T	A	A	A	T	A	A	A	T	A	C	T	T	T	A	A	T	T	C	A	C	A	C	T	A	G	T	C	T	A	A	A	C	A	C	A	T	T	C	A	G	G	T	C	A	T	?	?	?
Haplotype 47	?	?	?	?	?	A	A	A	A	A	T	A	A	A	T	A	C	T	T	C	A	A	T	T	C	A	C	A	C	T	A	G	T	C	T	A	A	A	C	A	C	A	T	T	C	A	G	G	T	C	A	T	T	A	A
Haplotype 48	?	?	?	?	?	?	A	A	G	G	T	A	A	A	T	A	C	T	T	T	A	A	T	T	C	A	C	A	C	T	A	G	T	C	T	A	A	A	C	A	C	A	T	T	C	A	G	G	T	C	A	T	T	A	A
Haplotype 49	?	?	?	?	A	C	T	A	A	G	T	T	A	A	T	A	C	T	T	T	A	A	T	T	C	A	C	A	C	T	A	G	T	C	T	A	A	A	C	A	C	A	T	T	C	A	G	G	T	C	A	T	T	A	A
Haplotype 50	T	G	T	G	A	C	T	A	A	A	T	A	A	A	T	A	C	T	T	T	A	A	T	T	C	A	C	A	C	T	A	G	T	C	T	A	A	G	C	A	C	A	T	T	C	A	G	G	T	C	A	T	T	A	?
Haplotype 51	T	G	T	G	A	C	T	A	A	A	T	A	A	A	T	A	C	T	T	T	A	A	T	T	C	A	C	A	C	T	A	G	T	C	T	A	A	A	C	A	C	A	T	T	C	A	T	G	T	C	A	T	T	?	?
Haplotype 52	T	G	T	G	A	C	T	A	A	A	T	A	A	A	T	A	C	T	T	T	A	A	T	T	C	A	C	A	C	T	A	G	T	C	C	A	A	A	C	A	T	A	T	T	C	A	T	G	T	C	A	T	T	A	?
Haplotype 53	T	G	T	G	A	C	T	A	A	A	T	A	A	A	T	A	C	T	T	T	A	A	T	T	C	A	C	A	T	T	A	G	T	C	C	A	A	A	C	A	T	A	T	T	C	A	T	G	T	C	A	T	T	A	?
Haplotype 54	?	?	?	?	A	C	T	A	A	A	T	A	A	A	T	A	C	T	T	T	A	A	T	T	C	A	C	A	C	T	A	G	T	C	T	A	A	A	C	A	C	A	A	T	C	A	G	G	C	C	A	T	T	A	A
Haplotype 55	?	G	T	G	A	C	T	A	A	A	T	A	A	A	T	A	C	T	T	T	A	A	T	T	C	A	C	A	C	T	A	G	T	C	T	A	A	A	C	A	C	A	T	T	T	A	G	G	T	C	A	T	T	A	A
Haplotype 56	T	G	T	G	A	C	T	A	A	A	T	A	A	A	T	A	C	T	T	T	A	A	T	T	C	A	C	A	C	T	A	G	T	C	T	A	A	A	C	A	C	A	T	T	T	A	G	G	T	C	A	C	C	?	?
Haplotype 57	?	?	?	?	?	C	T	A	A	A	T	A	C	A	T	A	C	T	T	T	G	A	T	T	C	A	C	A	C	T	A	G	T	C	T	A	A	A	C	A	C	A	T	T	T	A	G	G	T	C	A	T	T	A	C
Haplotype 58	?	G	T	G	A	C	T	A	G	A	T	A	A	A	T	A	C	T	T	T	A	A	T	T	C	A	C	A	C	T	A	G	T	C	T	A	A	A	C	A	C	A	T	T	T	A	G	G	T	C	A	T	T	A	A
Haplotype 59	?	G	T	A	A	C	T	A	G	A	T	A	A	A	T	A	C	T	T	T	A	A	T	T	C	A	C	A	C	T	A	G	T	C	T	A	A	A	C	A	C	A	T	T	T	A	G	G	T	C	A	T	T	A	A
Haplotype 60	?	?	T	A	A	C	A	G	G	A	T	A	A	A	T	A	C	T	T	T	A	A	T	T	C	A	C	A	C	T	A	G	T	C	T	A	A	A	C	A	C	A	T	T	T	A	G	G	T	C	A	T	T	A	A

Numbers in the columns indicate the position in the 472 bp amplified fragment.
